# Intergenerational social mobility and body mass index trajectories – A follow-up study from Finland

**DOI:** 10.1016/j.ssmph.2020.100723

**Published:** 2020-12-22

**Authors:** J. Salmela, T. Lallukka, N. Kanerva, O. Pietiläinen, O. Rahkonen, E. Mauramo

**Affiliations:** a Department of Public Health, University of Helsinki, P.O. Box 20, 00014, Helsinki, Finland; b Finnish Institute of Occupational Health, P.O. Box 18, 00032, Helsinki, Finland

**Keywords:** Body mass index, Education, Life-course approach, Social mobility, Trajectory analysis, Weight gain

## Abstract

Evidence remains unclear on how intergenerational social mobility is associated with body mass index (BMI) and its long-term changes. Our study identified BMI trajectories from middle to older age by intergenerational social mobility groups and stratified the analyses by gender and two birth cohorts (birth years 1940‒1947 and 1950–1962). We used questionnaire-based cohort data that consists of four survey phases: 2000–2002, 2007, 2012, and 2017. In Phase 1, participants were 40–60-year-old employees of the City of Helsinki, Finland. Our analytical sample consisted of 6,971 women and 1,752 men. Intergenerational social mobility was constructed based on self-reported parental and own education—both divided into high and low—yielding four groups: stable high socioeconomic position (SEP) (high-high), upward social mobility (low-high), downward social mobility (high-low), and stable low SEP (low-low). BMI was calculated from self-reported height and weight from all four phases. Using mixed-effects linear regression, we found increasing BMI trajectories in all four social mobility groups until the age of 65. Women and men with stable high SEP had lower BMI trajectories compared to those with stable low SEP. In the younger birth cohort, women with upward social mobility had a lower BMI trajectory than women with stable low SEP. Additionally, women and men with downward social mobility had higher BMI trajectories than those with stable high SEP. In the older birth cohort, however, the BMI trajectories of upward and downward social mobility groups were somewhat similar and settled between the BMI trajectories of stable high and stable low SEP groups. Our results indicate that the associations between intergenerational social mobility and BMI may depend on gender and birth cohort. Nevertheless, to reduce socioeconomic inequalities in unhealthy weight gain, obesity prevention actions that focus on people who are likely to remain in low SEP might be worthwhile.

## Highlights

• Few studies exist on social mobility and long-term body mass index (BMI) changes.
• Rising BMI trajectories were found in all social mobility groups.
• Stable low socioeconomic position (SEP) was related to higher BMI trajectories.
• Stable high SEP and upward social mobility may protect from excessive weight gain.
• Social mobility–BMI associations differed by gender and birth cohort.

## 1 Introduction

Unhealthy weight gain remains a great challenge for public health. Overweight and obesity increase the risk of morbidity and mortality (Nyberg et al., 2018; The Global BMI Mortality Collaboration, 2016), and a growing number of people worldwide are exposed to that burden (NCD Risk Factor Collaboration (NCD-RisC), 2017). Low socioeconomic position (SEP) is a known risk factor for obesity. Both low parental (Senese et al., 2009; Strand et al., 2012) and an individual's own SEP (C. Hart et al., 2008; Loman et al., 2013) have been associated with a higher adulthood body mass index (BMI), although there is limited evidence that either of them has a dominant role over the other (Laaksonen et al., 2004; Power et al., 2003). That has led researchers to investigate different life-course models to describe the associations between life-course SEP and BMI. For women, the accumulation of disadvantageous socioeconomic circumstances seems to have the most detrimental effect on BMI (Gustafsson et al., 2012; Heraclides & Brunner, 2010; Murray et al., 2011), whereas for men, some studies suggest that childhood SEP has the most critical impact on BMI (Murray et al., 2011). However, less attention has been paid to examining whether intergenerational social mobility—that is, the movement from parental SEP to one's own SEP—affects BMI and its long-term changes. In this study, we focus on the associations between intergenerational social mobility and BMI trajectories in the Finnish context.

Intergenerational social mobility is known to be relatively common in Finland as well as in the other Nordic welfare states (Causa & Johansson, 2010)—although, social disadvantage still remains intergenerationally inherited (Vauhkonen et al., 2017). Traditionally, social mobility is suggested to moderate SEP differences in health (C. L. Hart et al., 1998). The idea is that upward social mobility (i.e., individuals’ rising in the SEP hierarchy) would improve health compared to the situation of stable low SEP (i.e., remaining in low SEP over time). In turn, downward social mobility (i.e., deteriorating in SEP hierarchy) is considered to have negative effects on health compared to the situation of stable high SEP (i.e., remaining in high SEP over time). In addition, changes in SEP—both upward and downward—have been seen as stressful processes in themselves, which potentially affect health negatively: for example, through experienced social isolation or emotional imbalance experienced when moving to another social position or environment (Friedman, 2014). However, it has also been argued that social mobility as such would not be more burdensome to an individual than stable low SEP (Pollitt et al., 2005; Präg & Richards, 2019).

Stable low SEP has consistently been associated with a higher adulthood BMI, especially among women from high-income countries, but the evidence on upward and downward social mobility remains weak (Newton et al., 2017; Vieira et al., 2019). Mostly, the BMIs of people with upward and downward social mobility seem to be somewhere between the BMIs of stable high and stable low SEP groups (Hart et al., 2008; Langenberg et al., 2003). Among women, downward social mobility has been associated with higher BMI compared to those with stable high SEP (Boylan et al., 2014; Heraclides & Brunner, 2010). Additionally, upward social mobility has been associated with lower BMI compared to those with stable low SEP among women (Aitsi-Selmi et al., 2013; Ball & Mishra, 2006), and in some studies, among men as well (Langenberg et al., 2003). However, for men, there is no consensus that social mobility is associated with BMI (Vieira et al., 2019).

Several factors may explain the inconsistent previous findings on social mobility and BMI. First, there are different ways to define and measure social mobility. For example, both intra- and intergenerational social mobility—that is, whether the mobility occurs within a generation or between generations—have been examined. The reference group used and the number of measured time points vary between studies as well (Mishra et al., 2009). Second, the societal context of social mobility and population characteristics, such as gender, age, and country of residence, all affect results (Li et al., 2018; Malhotra et al., 2013; Padyab & Norberg, 2014). Third, upward and downward social mobility groups are often small, which can lead to statistically non-significant results (Langenberg et al., 2003; Padyab & Norberg, 2014). Lastly, the selection of the SEP measure that is used (e.g., education or income) may affect the results. Thus, more studies are needed to understand the link between social mobility and BMI and the modifying factors of these associations.

Social and cultural factors seem to have a major role in how SEP is transmitted between generations (Vauhkonen et al., 2017), but also, in how SEP is linked to BMI (Claassen et al., 2019). Therefore, it is possible that the social mobility–BMI associations differ over age depending on the temporal context of people's life-stage. Most studies have examined the associations between social mobility and BMI in early or middle adulthood (Aitsi-Selmi et al., 2013; Albrecht & Gordon-Larsen, 2014; Boylan et al., 2014), whereas less attention has been paid to long-term BMI changes until late adulthood. In addition, the associations found between social mobility and BMI are mainly based on studies that have used a BMI measure from only one time point in adulthood (Heraclides & Brunner, 2010; Murray et al., 2011; Padyab & Norberg, 2014). Some findings indicate that the associations between SEP and BMI may be stronger in later adulthood because of the accumulation of socioeconomic disadvantage over time (Gustafsson et al., 2012; Strand et al., 2012), but may also be strong in younger birth cohorts that have been exposed to obesogenic environments for a longer time (Bann et al., 2017).

The associations between intergenerational social mobility and BMI (and its changes) have not yet been examined in the Finnish context, whereas a few studies exist from the other Nordic countries. A Danish cohort study found that downward social mobility increased the risk of overweight and obesity among young female adults, compared to those with stable high SEP (Boylan et al., 2014). A Swedish study instead focused on intragenerational social mobility, and did not find it to be associated with BMI among 40–60-year-old adults (Padyab & Norberg, 2014). Previous studies by our research group have shown persistent and slightly widening socioeconomic inequalities in BMI among middle-aged and ageing Finns (Hiilamo et al., 2017; Salmela et al., 2020). This study proceeds from our previous findings and examines 1) whether intergenerational social mobility is associated with BMI trajectories from middle to older age (ages 40–77) and 2) whether these associations differ by gender and birth cohort.

## 2 Materials and methods

### 2.1 Study participants

All data were derived from the Helsinki Health Study cohort (Lahelma et al., 2013), which consists of four questionnaire surveys conducted in 2000–2002 (Phase 1), 2007 (Phase 2), 2012 (Phase 3), and 2017 (Phase 4). All 40-, 45-, 50-, 55-, and 60-year-old employees of the City of Helsinki, Finland (*n* = 13,344)—the largest employer in Finland with around 38,000 employees—were invited to participate in the Phase 1 survey. The response rate was 67% (*n* = 8,960). Similar questionnaires (Phases 2–4) were sent for those participants who responded in Phase 1, independent of their employment status at the time of each follow-up survey, yielding response rates of 83% (*n* = 7,332), 79% (*n* = 6,809), and 82% (*n* = 6,832), respectively. In this study, we excluded participants who were pregnant during Phase 1 (*n* = 23), had an outlier value in BMI (BMI<14 kg/m^2^ or BMI>60 kg/m^2^) (*n* = 4), had missing information on BMI in all phases (*n* = 30), or had missing information on parental or their own education (*n* = 180). On average, the included participants had BMI information in 3/4 time points. The final analytical sample consisted of 8,723 participants of which 80% (*n* = 6,971) were women, corresponding to the gender distribution in the public sector in Finland at large, among this age group.

### 2.2 Measures

#### 2.2.1 Intergenerational social mobility and BMI

The main exposure variable was *intergenerational social mobility,* which was constructed based on self-reported parental and the participant's own education. For parental education, matriculation or college examination or more was dichotomized into high (*n* = 1,862, 21%), and less than that into low education (*n* = 6,861, 79%). We inquired about both mother's and father's education, of which the higher one was used. For own education, university degree or equivalent was dichotomized into high (*n* = 2,280, 26%), and less than that into low education (*n* = 6,443, 74%). Consequently, the social mobility variable consisted of four groups: stable high SEP (*n* = 986, 11%), upward social mobility (*n* = 1,294, 15%), downward social mobility (*n* = 876, 10%), and stable low SEP (*n* = 5,567, 64%). *BMI*—the outcome variable—was calculated from self-reported height and weight (BMI in kg/m^2^ units) in each survey phase, and it was handled as a continuous variable.

#### 2.2.2 Covariates

Several covariates from Phase 1 were included in the supplementary analyses. Covariates were chosen based on previous studies that have proposed potential factors, such as health behaviors, economic circumstances, and mental health, to explain the life-course link between SEP and BMI (Gustafsson et al., 2012; Laaksonen et al., 2004; Novak et al., 2006). Those variables that were associated with both social mobility and BMI (*p* < 0.3) and did not have high mutual correlation (*r* < 0.7) were selected. *Marital status* was dichotomized into married or cohabiting and others. *Household income* was equivalized by dividing the typical monthly net income (7 income-level options) by household size that was weighted using the OECD equivalence scale (Hagenaars et al., 1994). Weighted household income was divided into quartiles, separately for women and men. *Economic difficulties* were measured with two questions that both included five response choices indicating the level of difficulties (Pearlin & Schooler, 1978): “How often do you not have enough money to buy the kind of food or clothing you or your family need?” and “How much difficulty do you have in meeting the payment of bills?“. We calculated a sum score and classified it into experiencing no (sum score 0), occasional (sum score 1–3), and frequent (sum score 4–8) economic difficulties.

*Fruit and vegetable consumption* was derived from a 20-item food frequency questionnaire and was dichotomized into daily and non-daily consumers. *Leisure-time physical activity (LTPA)* was based on a question of the volume (5 grades) and intensity (4 grades) of exercise during the past 12 months. We calculated weekly metabolic equivalent task (MET) hours (Ainsworth et al., 2000; Kujala et al., 1998), and divided participants into vigorously active (≥14 MET-hours/week including the two highest intensity grades), moderately active (≥14 MET-hours/week including the two lowest intensity grades), and inactive (<14 MET-hours/week). *Smoking* was dichotomized into non-smokers and smokers based on current smoking status (“Do you currently smoke? Yes/no”). *Sleep problems* were measured by a 4-item questionnaire of different insomnia symptoms, each with 6 response choices from not at all to 22–28 nights/month (Jenkins et al., 1988). We classified participants into having no, occasional (any symptoms in ≤14 nights/month), and frequent (any symptoms in >14 nights/month) sleep problems. *Physical and mental health functioning* were measured by the physical and mental component summary scores of the Short-Form 36 (SF-36) health questionnaire (Ware et al., 1994). The measurement scores were constructed to have a mean of 50 and standard deviation (SD) of 10 in the general population. Health functioning variables were treated as continuous measures where lower scores implyed poorer and higher scores better health functioning.

### 2.3 Statistical analyses

We used mixed-effects linear regression (mixed command in Stata 16, StataCorp LLC, College Station, TX, USA) to examine the associations between intergenerational social mobility and BMI trajectories over age. Mixed-effect models capture both fixed effects, which are the aspects of the model to define systematic features in the data (i.e., overall changes over age), and random effects, which are the model components that are allowed to vary between subjects (i.e., between-individual variance) (Van Dongen et al., 2004). We stratified all analyses by gender. To estimate whether the associations between social mobility and BMI trajectories differ by birth cohort, we divided the participants into younger (40–50-year-olds in Phase 1, birth years 1950–1962) and older (55–60-year-olds in Phase 1, birth years 1940–1947) birth cohorts, and performed sub-analyses separately for them. The interactions of social mobility*gender and social mobility*birth cohort were also tested among the whole analytical sample.

We first built a crude model (Model 1) which included the fixed effects of age, quadratic term of age, and social mobility; interaction between social mobility and age; and interaction between social mobility and quadratic term of age. Thus, these fixed effects allowed the model to capture intercepts and slopes in BMI over age for each social mobility group, considering curvilinearity in each BMI trajectory. Further, age and the quadratic term of age were also included as random effects to consider participant-specific intercepts and slopes in BMI over age. This curvilinear model proved to have the best fit after testing them against simpler models without interaction and quadratic terms. For the second model, we added marital status, household income, and economic difficulties as fixed effects (Model 2). For the full-adjusted model (Model 3), we further added health-related variables (fruit and vegetable consumption, leisure-time physical activity, sleep problems, smoking, and physical and mental health functioning) as fixed effects. Lastly, we calculated marginal means with 95% confidence intervals (CI) for BMI at each age and by social mobility groups.

Supplementary material describes the Stata commands used in the models, the model selection steps, and the equation of Model 1. Mixed-effects model statistics, including BMI estimates for the fixed-effect components with beta coefficients and standard errors, are shown in Tables S1–S3. As a sensitivity analysis, we performed complete case analyses which consisted of participants without any missing information on BMI, social mobility, and covariates (*n* = 3,988 for women, *n* = 870 for men). The patterns and orders of the BMI trajectories from these analyses were somewhat parallel with those of the main analyses (see Table S4 and Fig. S4–S5). Fig. S6 gives an overall picture of our study setting with temporal illustration of the associations between the measures.

## 3 Results

### 3.1 Characteristic of the study population

Most of the participants had stable low SEP: 65% of women and 57% of men (Table 1, Table 2). The participants from the younger birth cohort were more often downwardly than upwardly socially mobile. In Phase 1, mean BMI for women was 25.3 kg/m^2^ (SD, 4.4) and for men 26.4 kg/m^2^ (SD 3.9). Among women with stable high SEP and upward social mobility, higher household income, less economic difficulties, being a non-smoker, and better physical health functioning were more common compared to women with downward social mobility and stable low SEP (Table 1). A similar distribution could be seen among men, as well as being married or co-habiting, daily fruit and vegetable consumption, and being vigorously active in leisure-time were more common among stable high SEP and upward social mobility groups than among downward social mobility and stable low SEP groups (Table 2).

**Table 1:** Distributions by intergenerational social mobility groups among women from two birth cohorts, and cross tabulations for background covariates: the Helsinki Health Study Phase 1 survey (2000–2002).

	Social mobility group, *n* (%)					All, *n* (%)
	Stable high SEP	Upward mobility	Downward mobility	Stable low SEP	*p*-value, *X*^2^-test	
Total	723 (10)	994 (14)	691 (10)	4,563 (65)		6,971 (100)
Younger birth cohort^a^	462 (10)	648 (14)	494 (11)	2,878 (64)		4,482 (100)
Older birth cohort^a^	261 (10)	346 (14)	197 (8)	1,685 (68)		2,489 (100)
BMI (kg/m^2^), mean (SD)	24.1 (3.9)	24.4 (4.1)	24.9 (4.3)	25.8 (4.5)	<0.001^b^	25.3 (4.4)
Marital status					0.051	
Married/co-habiting	484 (67)	702 (71)	476 (69)	3,026 (67)		4,688 (67)
Others	238 (33)	289 (29)	214 (31)	1,523 (33)		2,264 (33)
Household income					<0.001	
Highest quartile	289 (41)	424 (44)	154 (23)	785 (18)		1,711 (25)
2nd highest	190 (27)	239 (25)	150 (22)	867 (20)		1,960 (29)
2nd lowest	167 (24)	211 (22)	199 (30)	1,383 (31)		1,446 (21)
Lowest quartile	63 (8.9)	94 (9.7)	170 (25)	1,384 (31)		1,652 (24)
Economic difficulties					<0.001	
No	434 (60)	634 (64)	370 (54)	2,074 (46)		3,512 (51)
Occasional	232 (32)	280 (28)	254 (37)	1,828 (41)		2,594 (38)
Frequent	54 (7.5)	71 (7.2)	64 (9.3)	592 (13)		781 (11)
Fruit and vegetable consumption					<0.001	
Daily	362 (50)	589 (59)	351 (51)	2,294 (50)		3,596 (52)
Non-daily	357 (50)	401 (41)	338 (49)	2,252 (50)		3,348 (48)
Leisure-time physical activity					<0.001	
Vigorously active	273 (38)	339 (34)	244 (35)	1,193 (26)		2,049 (30)
Moderately active	275 (39)	404 (41)	276 (40)	2,196 (49)		3,151 (46)
Inactive	166 (23)	246 (25)	169 (25)	1,132 (25)		1,713 (25)
Sleep problems					0.118	
No	78 (11)	123 (12)	93 (14)	600 (13)		894 (13)
Occasional	486 (68)	681 (69)	433 (63)	3,031 (67)		4,631 (67)
Frequent	154 (21)	186 (19)	159 (23)	895 (20)		1,394 (20)
Current smoker					<0.001	
No	618 (85)	870 (88)	503 (73)	3,339 (74)		5,330 (77)
Yes	105 (15)	117 (12)	182 (27)	1,170 (26)		1,574 (23)
Physical health functioning, mean score (SD)	50.7 (7.2)	50.9 (7.1)	49.4 (8.4)	48.0 (9.0)	<0.001^b^	48.8 (8.6)
Mental health functioning, mean score (SD)	49.8 (10.2)	50.8 (9.8)	51.1 (9.8)	52.1 (9.7)	<0.001^b^	51.6 (9.8)

a Birth years 1950–1962 for the younger birth cohort and 1940–1947 for the older birth cohort.

b *p*-value from one-way ANOVA test. Abbreviations: BMI = body mass index, SD = standard deviation, SEP = socioeconomic position.

**Table 2:** Distributions by intergenerational social mobility groups among men from two birth cohorts, and cross tabulations for background covariates: the Helsinki Health Study Phase 1 survey (2000–2002).

	Social mobility group, *n* (%)					All, *n* (%)
	Stable high SEP	Upward mobility	Downward mobility	Stable low SEP	*p*-value, *X*^2^-test	
Total	263 (15)	300 (17)	185 (11)	1,004 (57)		1,752 (100)
Younger birth cohort^a^	152 (15)	166 (16)	124 (12)	583 (57)		1,025 (100)
Older birth cohort^a^	111 (15)	134 (18)	61 (8)	421 (58)		727 (100)
BMI (kg/m^2^), mean (SD)	25.4 (3.4)	26.0 (3.4)	26.5 (4.1)	26.8 (4.0)	<0.001^b^	26.4 (3.9)
Marital status					<0.001	
Married/co-habiting	231 (88)	246 (83)	125 (68)	757 (76)		1,359 (78)
Others	31 (12)	50 (17)	58 (32)	244 (24)		383 (22)
Household income					<0.001	
Highest quartile	103 (39)	113 (38)	34 (18)	146 (15)		396 (23)
2nd highest	81 (31)	105 (35)	36 (20)	219 (22)		441 (25)
2nd lowest	50 (19)	51 (17)	52 (28)	303 (31)		456 (26)
Lowest quartile	28 (11)	31 (10)	62 (34)	321 (32)		442 (25)
Economic difficulties					<0.001	
No	173 (66)	197 (66)	81 (44)	455 (46)		906 (52)
Occasional	71 (27)	81 (27)	73 (40)	410 (41)		635 (36)
Frequent	17 (6.5)	20 (6.7)	30 (16)	132 (13)		199 (11)
Fruit and vegetable consumption					0.003	
Daily	78 (30)	104 (35)	49 (27)	241 (24)		472 (27)
Non-daily	183 (70)	196 (65)	135 (73)	758 (76)		1,272 (73)
Leisure-time physical activity					0.002	
Vigorously active	139 (53)	150 (50)	81 (45)	397 (40)		767 (44)
Moderately active	69 (26)	84 (28)	55 (30)	306 (31)		514 (30)
Inactive	55 (21)	65 (22)	45 (25)	289 (29)		454 (26)
Sleep problems					0.014	
No	35 (13)	54 (18)	31 (17)	192 (19)		312 (18)
Occasional	182 (69)	210 (70)	110 (60)	627 (63)		1,129 (65)
Frequent	45 (17)	35 (12)	41 (23)	177 (18)		298 (17)
Current smoker					<0.001	
No	206 (78)	245 (82)	123 (67)	690 (69)		1,264 (73)
Yes	57 (22)	54 (18)	60 (33)	307 (31)		478 (27)
Physical health functioning, mean score (SD)	51.9 (6.2)	52.1 (6.6)	49.9 (7.9)	49.6 (7.8)	<0.001^b^	50.4 (7.5)
Mental health functioning, mean score (SD)	50.1 (10.7)	51.6 (9.3)	50.3 (11.2)	51.9 (10.0)	0.037^b^	51.4 (10.1)

a Birth years 1950–1962 for the younger birth cohort and 1940–1947 for the older birth cohort.

b *p*-value from one-way ANOVA test. Abbreviations: BMI = body mass index, SD = standard deviation, SEP = socioeconomic position.

### 3.2 BMI trajectories by intergenerational social mobility groups

**Fig. 1:**
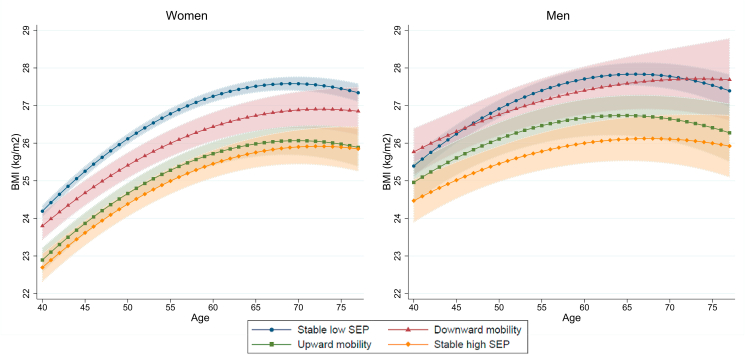
Body mass index (BMI) trajectories by intergenerational social mobility groups over age among women and men. Unadjusted models (Model 1): predictive margins—that is, mean BMIs for social mobility groups at each age year—with 95% confidence intervals from mixed-effects linear regression. Abbreviations: SEP = socioeconomic position.

Fig. 1 shows unadjusted models for BMI trajectories by intergenerational social mobility groups, among women and men. Overall, rising BMI trajectories were found in all groups until the age of 68 among women and until the age of 65 among men. At the age of 40, the mean BMIs of social mobility groups were 22.7–24.2 among women, whereas among men, they were 24.5–25.8. During older age, women reached somewhat similar BMI levels as men (mean BMI 25.8–27.3 for women and 25.9–27.7 for men at the age of 77). No differences were found in the shapes of BMI trajectories (i.e., curvilinear age effect in BMI) when comparing other social mobility groups to the stable high SEP group among women. However, among men, a curvilinear difference was found (*p* < 0.05) between stable low and stable high SEP groups.

Women with stable high SEP and upward social mobility had lower BMI trajectories than women with stable low SEP over age (Fig. 1). Additionally, women with stable high SEP had lower BMI trajectories than women with downward social mobility until the age of 71 (mean BMI 25.9, 95% CI 25.4–26.4, vs. mean BMI 26.9, 95% CI 26.4–27.4). Among men, mean BMI was lower in the stable high SEP group (24.5, 95% CI 23.9–25.0) compared to the groups of downward social mobility (25.8, 95% CI 25.1–26.4) and stable low SEP (25.4, 95% CI 25.1–25.7) at the age of 40. These differences remained over age—technically, until the age of 73 for the downward social mobility group. In contrast to women, men with downward social mobility had the highest mean BMI before the age of 47 and after the age of 71.

**Fig. 2:**
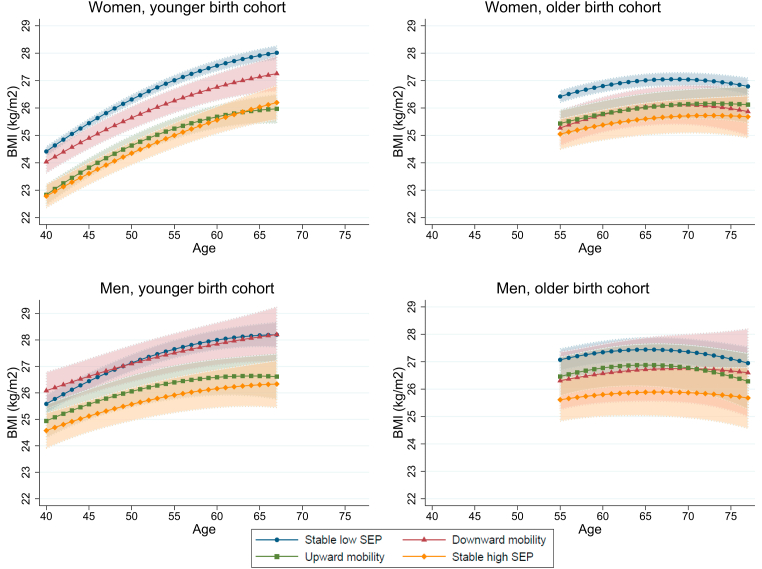
Body mass index (BMI) trajectories by intergenerational social mobility groups over age, stratified by gender and birth cohort. Birth years 1950–1962 for younger and 1940–1947 for older birth cohort. Unadjusted models (Model 1): predictive margins—that is, mean BMIs for social mobility groups at each age year—with 95% confidence intervals from mixed-effects linear regression. Abbreviations: SEP = socioeconomic position.

The associations between social mobility groups and BMI trajectories differed by birth cohort (Fig. 2). At the age of 60, for example, mean BMIs of social mobility groups were higher among the younger birth cohort compared to the older cohort (25.6–27.5 vs. 25.4–26.8 for women, and 26.2–28.0 vs. 25.8–27.3 for men). Overall, the results of the younger cohort corresponded to the results found when examining the birth cohorts together. For men, widening BMI differences between stable high and low SEP groups became even more evident (mean BMI difference 1.0 units at the age of 40 and 1.9 units at the age of 67). However, among the participants from the older birth cohort, the BMI trajectories of upward and downward social mobility groups followed similar trends over older age, and they were not higher compared to the stable high SEP groups. For women from the older cohort, BMI trajectory differences were found only between stable high and low SEP groups. Similarly, among men from the older cohort, BMI trajectory differences were found only between stable high and low SEP groups—with the phenomenon ceasing after the age of 71 (mean BMI 25.9, 95% CI 24.9–26.8, vs. mean BMI 27.3, 95% CI 26.8–27.8).

Adjustment of other socioeconomic and health-related factors narrowed the social mobility group differences (Fig. S1). Frequent economic difficulties, lighter physical activity, and poorer physical health functioning raised the BMI trajectory levels among both genders (*p* < 0.05), whereas an opposite effect was found for smoking (Table S1). Among women, BMI trajectory differences between stable high and low SEP groups remained over age in both birth cohorts (except last few years in older birth cohort) after adjustments (Fig. S2). Men with downward social mobility and stable low SEP remained having a higher BMI trajectory than men with stable high SEP in some ages in the younger birth cohort (Fig. S3). Mostly, however, no social mobility–BMI trajectory associations were found for men after adjustments.

## 4 Discussion

We examined the associations between intergenerational social mobility and BMI trajectories among 40‒77-year-old Finnish women and men from two birth cohorts (birth years 1940–1947 and 1950–1962). Overall, rising BMI trajectories were found in all social mobility groups until the age of 65. BMI differences between stable high and low SEP groups were somewhat constant over age, corresponding to the Nordic inequality trends in overweight and obesity from recent decades (Magnusson et al., 2014). In Finland, SEP disparities in BMI have remained somewhat stable during the latest decades while mean BMI is increasing among all adults, both low and high SEP (Prättälä et al., 2012). We found some widening differences between stable low and high SEP groups among men from the younger birth cohort, but these differences were not greater at any age compared to the differences found among women. Our results support the strong evidence from previous studies where stable low SEP has been associated with higher BMI, particularly among women in high-income countries (Newton et al., 2017; Vieira et al., 2019). Vulnerability to weight gain among constantly disadvantaged women has been explained, for example, by a greater stress response to social disadvantage and greater weight-related social and occupational discrimination compared to men (Pudrovska et al., 2014).

The existing literature remains ambiguous about the influence of upward and downward social mobility on BMI. Our results support the findings of the detrimental impact of downward social mobility on BMI and health in general (Boylan et al., 2014; Krzyanowska & Mascie-Taylor, 2011; Melchior et al., 2006). Additionally, we found upward social mobility to be associated with lower BMI trajectories than of those with stable low SEP among women, which is consistent with previous findings (Aitsi-Selmi et al., 2013; Ball & Mishra, 2006). Contrary to some previous studies (Heraclides & Brunner, 2010; Savitsky et al., 2017), we did not find upward social mobility to be associated with a higher BMI trajectory compared to stable high SEP, neither among women nor men. However, the comparison between studies is, in some cases, rather complex because the reference groups vary: both stable high SEP (Savitsky et al., 2017), stable low SEP (Heraclides & Brunner, 2010), and social immobility in general (Krzyanowska & Mascie-Taylor, 2011) have been used in the between-group comparisons. In addition, not all studies have stratified the analyses by gender (Albrecht & Gordon-Larsen, 2014; Heraclides & Brunner, 2010).

Evident birth cohort differences were observed in how upward and downward social mobility were associated with the BMI trajectories. For participants from the younger cohort, one's own SEP seemed to largely define their BMI trajectories. Men with downward social mobility had a higher BMI trajectory than of those with stable high SEP; this is contrary to studies that have suggested men's inequalities in BMI to emerge in childhood, and later-life SEP to have less impact on their BMI (Langenberg et al., 2003; Murray et al., 2011; Padyab & Norberg, 2014). A similar association was found among women from the younger birth cohort. However, for women and men from the older birth cohort, upward and downward social mobility groups settled between the BMI trajectories of stable high and low SEP. This supports the studies in which the contribution of socioeconomic accumulation to BMI has been shown to become more visible over age (Gustafsson et al., 2012), and where social mobility is argued to diminish health inequalities (Langenberg et al., 2003; Pollitt et al., 2005). A longer follow-up time with the participants from the younger cohort would reveal whether these birth cohort differences persist in older age as well.

Although the intergenerational social mobility measure that we used captured social mobility before the follow-up (and so, before the BMI trajectories), we cannot verify causality between social mobility and BMI trajectories. Our previous study showed that most of the weight gain occurred before middle adulthood, and that lower childhood SEP was associated with higher BMI trajectories (Salmela et al., 2019). Thus, SEP differences in BMI have probably existed long before the follow-up. In general, a social causation hypothesis (i.e., SEP affects health) is better supported than a health-related selection hypothesis (i.e., health affects SEP) (Dahl, 1996). Health-related selection has been assumed to concern younger ages, whereas during adulthood, social causation further increases the existing health inequalities (Elovainio et al., 2011). Additionally, health-related social mobility usually concerns only a small amount of people, and thus, its impact on narrowing health inequalities have been seen to be only moderate (Dahl, 1996; Rahkonen et al., 1997).

Using education as an SEP measure is appropriate in our study setting. Since the youngest participants were 40 years old in Phase 1, we can assume that most of the participants (and their parents) completed their highest education before the follow-up; thus, further social mobility after Phase 1 is not probable among the participants. Although different SEP measures are not interchangeable because they capture different aspects of SEP (Braveman et al., 2005), the selection between education and occupation as an SEP measure in high-income Western countries has not substantially affected the findings of the SEP inequalities in BMI (McLaren, 2007; Vieira et al., 2019). Education encompasses not only material aspects of SEP but also non-material aspects, such as knowledge, literacy, and cognitive capacity (Braveman et al., 2005; Galobardes et al., 2006). Thus, it probably accurately captures the psychosocial aspects, such as perceived stress or exposure to neighborhood perceptions, that are assumed to partly explain SEP differences in BMI (Claassen et al., 2019).

We found that economic circumstances and health-related factors explained part, but not all, of the social mobility group differences in BMI trajectories. In previous studies, persistent socioeconomic inequalities in weight gain and obesity have been explained by multiple factors, such as prolonged financial strain (Li, 2015; Salmela et al., 2020), learned unhealthy behaviors (Albrecht & Gordon-Larsen, 2014; Novak et al., 2006), and psychosocial factors (Chung et al., 2020; Claassen et al., 2019). However, none of them likely has a separate role in the process (Umberson et al., 2010); for example, health behaviors are argued to be insufficient in explaining—and thus, reducing—socioeconomic inequalities in obesity (Gustafsson et al., 2012; Savitsky et al., 2017). Instead, sociocultural resources, such as habits, behaviors, and attitudes, have a major role in how SEP is transmitted between generations (Vauhkonen et al., 2017). These resources probably reflect the persistent inequalities in BMI as well. Nevertheless, more studies are needed to evaluate these mechanisms.

In the same way that we cannot fully distinguish different life-course models from each other, the effects of age, period, and cohort operate simultaneously in the associations between life-course SEP and BMI (Hallqvist et al., 2004; Keyes et al., 2010). Although we focused here on the roles of age and birth cohort in the relationship between social mobility and BMI trajectories, the period effects probably have a notable contribution to the results due to widespread changes toward obesogenic environments in recent decades (Clarke et al., 2009). Higher and steeper BMI trajectories in the younger birth cohort were not surprising since these participants have been affected by an obesogenic environment earlier in their life than the participants from the older cohort (Keyes et al., 2010). Several studies have found greater and widening inequalities in BMI among younger birth cohorts in Western countries (Bann et al., 2017; Hayes et al., 2019; Norris et al., 2020), although a substantial increase in BMI among people with high SEP has been indicated to diminish these differences as well (Clarke et al., 2009). In addition to changes in the physical environment, educational development has been rapid during our study participants’ lifetime (Statistics Finland, 2007). These temporal changes together may explain why the social mobility group differences in BMI trajectories varied between younger and older birth cohorts: the exposure time to these changes has been different.

A major strength of this study is the prospective cohort study setting with four identical survey phases, comprising participants with a large age scale (22 years) in every phase. That enabled us to examine social mobility group differences in long-term BMI changes until late adulthood, which to our knowledge has not been done before. Additionally, the age variety enabled us to examine birth cohort differences in social mobility–BMI trajectory associations, which previous studies have paid little attention to. Our study sample represents the target population well: midlife and ageing Finns with municipal employment backgrounds; the response rates in every survey phase were at least satisfactory, and nonparticipation does not seriously bias the results (Laaksonen et al., 2008; Lahelma et al., 2013). Older employees and those with a higher occupational class and income were slightly overrepresented than in the target population (Laaksonen et al., 2008).

We measured intergenerational social mobility in relative terms because of the substantial rising in educational levels during the 20th century in Finland (Statistics Finland, 2007). Relative social mobility uses different cut-off points for parental and one's own SEP measures, whereas absolute social mobility uses similar classifications for parental and own SEP. Relative social mobility has been argued to be a more appropriate way to capture the impact of social mobility on health and health-related behavior compared to absolute mobility, which does not take into account societal changes in SEP (Galobardes et al., 2006; Gugushvili et al., 2019). However, that choice also led to a high number of participants with stable low SEP and a small number of participants with upward social mobility. These social mobility group sizes, though, should not be mixed to illustrate the distribution of absolute mobility among the study participants (in which case, e.g., upward social mobility would be more common).

Some limitations must be considered as well. First, although our sample represents the target population well, it is not representative of the general Finnish population: for instance, people from the private sector or outside the labor market were not included, and all the participants lived during Phase 1 in the Capital area of Southern Finland. Second, intergenerational social mobility was determined conventionally by combining two time points, social positions of origin and destination, yielding four distinct social mobility groups. Using this conventional approach to examine social mobility has been criticized for its simplicity, inability to take into account the timing of mobility, and inability to distinguish mobility effects from origin and destination effects (Li et al., 2018; van der Waal et al., 2017). Three or more SEP measurement points could provide a more comprehensive picture of the real SEP trajectories, but that would lead to small social mobility groups and further complicate the analyses and their interpretation (Pollitt et al., 2005). Third, the variables were based on self-reports, which can be biased: recall of parental education can be inaccurate, and BMI is probably underestimated (Connor Gorber et al., 2007). Lastly, missing data have some minor impacts on the results, according to our sensitivity analyses; BMI trajectories are probably slightly higher and the social mobility group differences greater in the population than these results showed since the opposite effect was found among participants with complete data in all study variables (see Fig. S4–S5).

## 5 Conclusions

We found persistent inequalities between stable high and low SEP groups in BMI trajectories among Finnish women and men from two birth cohorts (1940–1947 and 1950–1962). Downward social mobility was associated with a higher BMI trajectory, particularly in the younger birth cohort, whereas people with upward social mobility did not have a higher BMI trajectory compared to those with stable high SEP. Although stable low SEP seems to have the most detrimental impact on weight development, especially among women, our results provide some positive insight on how transitioning from low to high SEP may protect against excessive weight gain. Because the number of people with upward social mobility was relatively low, however, the impact of upward social mobility on tackling unhealthy weight gain at a societal level remains slight. Preventive efforts should target the population groups which tend to remain or end up in low SEP in adulthood. Since the temporal sociocultural context probably has a major contribution to how social mobility affects long-term BMI changes, future studies should pay more attention to clarify how age, period and cohort modify these associations.

## CRediT authorship contribution statement

**J. Salmela:** Conceptualization, Data curation, Formal analysis, Funding acquisition, Investigation, Methodology, Software, Visualization, Writing - original draft, Writing - review & editing. **T. Lallukka:** Conceptualization, Funding acquisition, Supervision, Writing - review & editing. **N. Kanerva:** Conceptualization, Supervision, Writing - review & editing. **O. Pietiläinen:** Data curation, Methodology, Writing - review & editing. **O. Rahkonen:** Conceptualization, Funding acquisition, Project administration, Resources, Writing - review & editing. **E. Mauramo:** Conceptualization, Supervision, Writing - review & editing.

## Declaration of competing interest

The authors have no conflicts of interest.
