# Supplementary material for: Intergenerational social mobility and body mass index trajectories – A follow-up study from Finland
Source: SSM Popul Health. 2020 Dec 22;13:100723. doi: 10.1016/j.ssmph.2020.100723 (PMC7770483; doi:10.1016/j.ssmph.2020.100723)
Supplement: Multimedia component 1 [file mmc1.docx]

Supplementary material

STATA codes for mixed-effects linear regression models

**Model 1**

mixed c.BMI i.SocialMobility##c.Age##c.Age || ID: Age Age^2^

**Model 2**

mixed c.BMI i.SocialMobility##c.Age##c.Age i.MaritalStatus i.HouseholdIncome i.EconomicDifficulties || ID: Age Age^2^

**Model 3**

mixed c.BMI i.SocialMobility##c.Age##c.Age i.MaritalStatus i.HouseholdIncome i.EconomicDifficulties i.F&V i.LTPA i.SleepProblems i.Smoking i.PhysicalHealthFunctioning i.MentalHealthFunctioning || ID: Age Age^2^

Model selection steps

**1. Selection of the structure of the random effects using “full-loaded” crude model**

(1) mixed c.BMI i.SocialMobility##c.Age##c.Age || ID: Age Age^2^

(2) mixed c.BMI i.SocialMobility##c.Age##c.Age || ID: Age

Likelihood-ratio test (assumption: 2 nested in 1): *p* < 0.001 for women and men.

**2. Selection of the final crude model by comparing the “full-loaded” crude model to the simpler one**

(1) Different linear and curvilinear age effects for social mobility groups:

mixed c.BMI i.SocialMobility##c.Age##c.Age || ID: Age Age^2^

(2) Same linear and curvilinear age effects for social mobility groups:

mixed c.BMI i.SocialMobility c.Age##c.Age || ID: Age Age^2^

Likelihood-ratio test (assumption: 2 nested in 1): *p* = 0.0790 for women and *p* = 0.0253 for men. Despite a non-significant *p*-value from the likelihood-ratio test for women—supporting the selection of the simpler model—we selected modeling different linear and curvilinear age effects for social mobility groups for both women and men because, for men, that proved to fit the model better.

Equation of the crude model (Model 1)

BMI_ij_ = β_0_ + β_1_ × Age_ij_ + β_2_ × Age^2^_ij_ + β_3_ × SMGP1_i_ + β_4_ × SMGP2_i_ + β_5_ × SMGP3_i_

+ β_6_ × Age_ij_ × SMGP1_i_ + β_7_ × Age_ij_ × SMGP2_i_ + β_8_ × Age_ij_ × SMGP3_i_

Fixed effects

+ β_9_ × Age^2^_ij_ × SMGP1_i_ + β_10_ × Age^2^_ij_ × SMGP2_i_ + β_11_ × Age^2^_ij_ × SMGP3_i_

+ *u*_0i_ + *u*_1i_ × Age_ij_ + *u*_2i_ × Age^2^_ij_ + ε_ij_

Random effects

Where (fixed effects):

- β_0_ = Intercept
- β_1_ = Age
- β_2_ = Age^2^
- β_3_ = SMGP1
- β_4_ = SMGP2
- β_5_ = SMGP3
- β_6_ = Age × SMGP1
- β_7_ = Age × SMGP2
- β_8_ = Age × SMGP3
- β_9_ = Age^2^ × SMGP1
- β_10_ = Age^2^ × SMGP2
- β_11_ = Age^2^ × SMGP3

(random effects):

- *u*_0i_ = Individual-specific intercept
- *u*_1i_ = Linear effect of age for individual i
- *u*_2i_ = Quadratic effect of age for individual i

(residuals):

- ε_ij_ = Residuals for observation at age j on individual i

Note: Intergenerational social mobility variable was classified as stable high SEP = 0, upward social mobility = 1, downward social mobility = 2, and stable low SEP = 3. Stable high SEP was considered the reference group, thus, the social mobility variables that have been left in the equation model are social mobility groups (SMGP) 1, 2, and 3.

Supplementary tables

**Table S1.** Body mass index estimates from mixed-effects linear regression for fixed-effect components among women and men. Beta coefficients with standard errors in parentheses are shown.

|  | **Women** | | | **Men** | | |
| --- | --- | --- | --- | --- | --- | --- |
|  | **Model 1 ^a^** | **Model 2 ^b^** | **Model 3 ^c^** | **Model 1 ^a^** | **Model 2 ^b^** | **Model 3 ^c^** |
| **Fixed effects, β (SE)** |  |  |  |  |  |  |
| Age | 0.45 (0.05)* | 0.46 (0.05)* | 0.44 (0.05)* | 0.30 (0.07)* | 0.31 (0.07)* | 0.31 (0.07)* |
| Age^2^ | -0.003 (0.000)* | -0.003 (0.000)* | -0.003 (0.000)* | -0.002 (0.001)* | -0.002 (0.001)* | -0.002 (0.001)* |
| Social mobility (ref. stable high SEP) |  |  |  |  |  |  |
| Upward mobility | -1.04 (1.71) | -0.79 (1.73) | -1.09 (1.75) | -1.71 (2.71) | -1.96 (2.75) | -1.50 (2.74) |
| Downward mobility | 1.79 (1.82) | 2.00 (1.84) | 1.45 (1.86) | 2.18 (3.02) | 1.65 (3.05) | 1.50 (3.08) |
| Stable low SEP | -1.22 (1.39) | -1.39 (1.41) | -1.75 (1.43) | -4.10 (2.26) | -4.15 (2.28) | -3.71 (2.29) |
| Social mobility (ref. stable high SEP)*Age |  |  |  |  |  |  |
| Upward mobility | 0.05 (0.06) | 0.04 (0.06) | 0.05 (0.06) | 0.09 (0.09) | 0.09 (0.09) | 0.07 (0.09) |
| Downward mobility | -0.02 (0.06) | -0.04 (0.07) | -0.02 (0.07) | -0.04 (0.11) | -0.03 (0.11) | -0.03 (0.11) |
| Stable low SEP | 0.10 (0.05)* | 0.10 (0.05)* | 0.10 (0.05)* | 0.18 (0.08)* | 0.18 (0.08)* | 0.15 (0.08) |
| Social mobility (ref. stable high SEP)*Age^2^ |  |  |  |  |  |  |
| Upward mobility | 0.000 (0.001) | 0.000 (0.001) | 0.000 (0.001) | -0.001 (0.001) | -0.001 (0.001) | -0.001 (0.001) |
| Downward mobility | 0.000 (0.001) | 0.000 (0.001) | 0.000 (0.001) | 0.000 (0.001) | 0.000 (0.001) | 0.000 (0.001) |
| Stable low SEP | -0.001 (0.000)* | -0.001 (0.000) | -0.001 (0.000) | -0.001 (0.001)* | -0.001 (0.001)* | -0.001 (0.001) |
| Marital status (ref. married/cohabiting) |  |  |  |  |  |  |
| Others |  | -0.19 (0.12) | -0.19 (0.12) |  | -0.02 (0.23) | -0.06 (0.23) |
| Household income  (ref. highest quartile) |  |  |  |  |  |  |
| 2^nd^ highest |  | 0.26 (0.16) | 0.29 (0.16) |  | -0.11 (0.27) | 0.06 (0.27) |
| 2^nd^ lowest |  | 0.35 (0.16)* | 0.42 (0.16)* |  | 0.17 (0.28) | 0.11 (0.28) |
| Lowest quartile |  | 0.42 (0.18)* | 0.40 (0.17)* |  | 0.31 (0.30) | 0.30 (0.29) |
| Economic difficulties  (ref. no) |  |  |  |  |  |  |
| Occasional |  | 0.83 (0.12)* | 0.80 (0.12)* |  | 0.39 (0.21) | 0.32 (0.21) |
| Frequent |  | 1.03 (0.19)* | 0.85 (0.19)* |  | 1.39 (0.32)* | 1.23 (0.32)* |
| Fruit and vegetable consumption  (ref. daily) |  |  |  |  |  |  |
| Non-daily |  |  | 0.16 (0.11) |  |  | 0.81 (0.21)* |
| Leisure-time physical activity (ref. vigorously active) |  |  |  |  |  |  |
| Moderately active |  |  | 1.00 (0.13)* |  |  | 0.88 (0.22)* |
| Inactive |  |  | 2.19 (0.15)* |  |  | 1.62 (0.24)* |
| Sleep problems (ref. no) |  |  |  |  |  |  |
| Occasional |  |  | -0.25 (0.17) |  |  | -0.34 (0.26) |
| Frequent |  |  | -0.32 (0.22) |  |  | 0.10 (0.36) |
| Current smoker (ref. no) |  |  |  |  |  |  |
| Yes |  |  | -0.34 (0.13)* |  |  | -0.71 (0.21)* |
| Physical health functioning (cont.) |  |  | -0.08 (0.01)* |  |  | -0.07 (0.01)* |
| Mental health functioning (cont.) |  |  | 0.02 (0.01)* |  |  | 0.003 (0.01) |
| Intercept | 9.75 (1.28)* | 8.87 (1.29)* | 12.17 (1.43)* | 16.14 (1.99)* | 15.55 (2.02)* | 18.44 (2.29)* |

^a^ Unadjusted model. ^b^ Model 1 + marital status, household income, and economic difficulties. ^c^ Model 2 + fruit and vegetable consumption, leisure-time physical activity, sleep problems, smoking, physical health functioning, and mental health functioning. Abbreviations: SE = standard error, SEP = socioeconomic position. * *p*-value < 0.05.

**Table S2.** Body mass index estimates from mixed-effects linear regression for fixed-effect components among women from the younger (birth years 1950–1962) and older (birth years 1940‒1947) birth cohorts. Beta coefficients with standard errors in parentheses are shown.

|  | **Women, younger birth cohort** | | | **Women, older birth cohort** | | |
| --- | --- | --- | --- | --- | --- | --- |
|  | **Model 1 ^a^** | **Model 2 ^b^** | **Model 3 ^c^** | **Model 1 ^a^** | **Model 2 ^b^** | **Model 3 ^c^** |
| **Fixed effects, β (SE)** |  |  |  |  |  |  |
| Age | 0.32 (0.08)* | 0.33 (0.08)* | 0.32 (0.08)* | 0.32 (0.17) | 0.36 (0.17)* | 0.27 (0.18) |
| Age^2^ | -0.002 (0.001)* | -0.002 (0.001)* | -0.002 (0.001)* | -0.002 (0.001) | -0.003 (0.001) | -0.002 (0.001) |
| Social mobility (ref. stable high SEP) |  |  |  |  |  |  |
| Upward mobility | -4.82 (2.69) | -4.63 (2.71) | -4.52 (2.71) | 0.02 (7.10) | 1.53 (7.26) | -1.16 (7.46) |
| Downward mobility | -0.26 (2.88) | -0.33 (2.90) | -0.99 (2.92) | -7.66 (8.15) | -6.54 (8.25) | -8.81 (8.54) |
| Stable low SEP | -2.80 (2.19) | -2.89 (2.19) | -3.02 (2.19) | -3.42 (5.85) | -2.47 (5.98) | -4.35 (6.20) |
| Social mobility (ref. stable high SEP)*Age |  |  |  |  |  |  |
| Upward mobility | 0.20 (0.10) | 0.19 (0.10) | 0.19 (0.10) | 0.01 (0.22) | -0.04 (0.23) | 0.04 (0.23) |
| Downward mobility | 0.06 (0.11) | 0.06 (0.11) | 0.08 (0.11) | 0.25 (0.25) | 0.21 (0.26) | 0.28 (0.27) |
| Stable low SEP | 0.17 (0.08)* | 0.16 (0.08) | 0.15 (0.08) | 0.16 (0.18) | 0.12 (0.19) | 0.17 (0.19) |
| Social mobility (ref. stable high SEP)*Age^2^ |  |  |  |  |  |  |
| Upward mobility | -0.002 (0.001)* | -0.002 (0.001) | -0.002 (0.001) | 0.000 (0.002) | 0.000 (0.002) | 0.000 (0.002) |
| Downward mobility | -0.001 (0.001) | -0.001 (0.001) | -0.001 (0.001) | -0.002 (0.002) | -0.002 (0.002) | -0.002 (0.002) |
| Stable low SEP | -0.002 (0.001) | -0.001 (0.001) | -0001 (0.001) | -0.001 (0.001) | -0.001 (0.001) | -0.001 (0.002) |
| Marital status (ref. married/cohabiting) |  |  |  |  |  | -0.29 (0.21) |
| Others |  | -0.07 (0.15) | -0.03 (0.15) |  | -0.40 (0.21) |  |
| Household income  (ref. highest quartile) |  |  |  |  |  |  |
| 2^nd^ highest |  | 0.28 (0.21) | 0.27 (0.21) |  | 0.30 (0.26) | 0.27 (0.26) |
| 2^nd^ lowest |  | 0.35 (0.20) | 0.39 (0.20) |  | 0.34 (0.28) | 0.16 (0.27) |
| Lowest quartile |  | 0.52 (0.22)* | 0.43 (0.22)* |  | 0.07 (0.29) | -0.03 (0.29) |
| Economic difficulties  (ref. no) |  |  |  |  |  |  |
| Occasional |  | 0.77 (0.15)* | 0.72 (0.15)* |  | 0.93 (0.21)* | 0.86 (0.21)* |
| Frequent |  | 1.11 (0.22)* | 0.86 (0.23)* |  | 0.51 (0.34) | 0.35 (0.34) |
| Fruit and vegetable consumption  (ref. daily) |  |  |  |  |  |  |
| Non-daily |  |  | 0.12 (0.14) |  |  | 0.02 (0.19) |
| Leisure-time physical activity (ref. vigorously active) |  |  |  |  |  |  |
| Moderately active |  |  | 0.97 (0.15)* |  |  | 1.31 (0.23)* |
| Inactive |  |  | 1.99 (0.18)* |  |  | 2.82 (0.27)* |
| Sleep problems (ref. no) |  |  |  |  |  |  |
| Occasional |  |  | -0.36 (0.20) |  |  | -0.11 (0.29) |
| Frequent |  |  | -0.57 (0.27)* |  |  | 0.06 (0.36) |
| Current smoker (ref. no) |  |  |  |  |  |  |
| Yes |  |  | -0.31 (0.16) |  |  | -0.67 (0.24)* |
| Physical health functioning (cont.) |  |  | -0.10 (0.01)* |  |  | -0.08 (0.01)* |
| Mental health functioning (cont.) |  |  | 0.01 (0.01) |  |  | 0.04 (0.01)* |
| Intercept | 12.99 (2.01)* | 12.04 (2.02)* | 16.19 (2.15)* | 14.07 (5.43)* | 12.71 (5.55)* | 16.51 (5.84)* |

^a^ Unadjusted model. ^b^ Model 1 + marital status, household income, and economic difficulties. ^c^ Model 2 + fruit and vegetable consumption, leisure-time physical activity, sleep problems, smoking, physical health functioning, and mental health functioning. Abbreviations: SE = standard error, SEP = socioeconomic position. * *p*-value < 0.05.

**Table S3.** Body mass index estimates from mixed-effects linear regression for fixed-effect components among men from the younger (birth years 1950–1962) and older (birth years 1940‒1947) birth cohorts. Beta coefficients with standard errors in parentheses are shown.

|  | **Men, younger birth cohort** | | | **Men, older birth cohort** | | |
| --- | --- | --- | --- | --- | --- | --- |
|  | **Model 1 ^a^** | **Model 2 ^b^** | **Model 3 ^c^** | **Model 1 ^a^** | **Model 2 ^b^** | **Model 3 ^c^** |
| **Fixed effects, β (SE)** |  |  |  |  |  |  |
| Age | 0.28 (0.12)* | 0.33 (0.13)* | 0.38 (0.13)* | 0.27 (0.22) | 0.24 (0.22) | 0.19 (0.22) |
| Age^2^ | -0.002 (0.001) | -0.002 (0.001)* | -0.003 (0.001)* | -0.002 (0.002) | -0.002 (0.002) | -0.001 (0.002) |
| Social mobility (ref. stable high SEP) |  |  |  |  |  |  |
| Upward mobility | -1.95 (4.57) | -1.05 (4.64) | 0.48 (4.63) | -7.53 (9.53) | -8.75 (9.58) | -10.57 (9.63) |
| Downward mobility | 2.67 (4.83) | 3.19 (4.89) | 3.61 (4.97) | -1.08 (12.91) | -2.62 (12.96) | -8.92 (13.29) |
| Stable low SEP | -4.02 (3.74) | -3.16 (3.77) | -0.73 (3.80) | -4.48 (7.98) | -5.85 (8.05) | -8.41 (8.17) |
| Social mobility (ref. stable high SEP)*Age |  |  |  |  |  |  |
| Upward mobility | 0.09 (0.17) | 0.05 (0.18) | -0.003 (0.18) | 0.27 (0.30) | 0.31 (0.30) | 0.36 (0.30) |
| Downward mobility | -0.05 (0.19) | -0.08 (0.19) | -0.10 (0.19) | 0.05 (0.40) | 0.09 (0.41) | 0.27 (0.42) |
| Stable low SEP | 0.18 (0.14) | 0.14 (0.14) | 0.04 (0.14) | 0.19 (0.25) | 0.22 (0.25) | 0.29 (0.25) |
| Social mobility (ref. stable high SEP)*Age^2^ |  |  |  |  |  |  |
| Upward mobility | -0.001 (0.0002) | -0.001 (0.002) | 0.000 (0.002) | -0.002 (0.002) | -0.002 (0.002) | -0.003 (0.002) |
| Downward mobility | 0.001 (0.002) | 0.001 (0.002) | 0.001 (0.002) | 0.000 (0.003) | -0.001 (0.003) | -0.002 (0.003) |
| Stable low SEP | -0.001 (0.001) | -0.001 (0.001) | 0.000 (0.001) | -0.002 (0.002) | -0.002 (0.002) | -0.002 (0.002) |
| Marital status (ref. married/cohabiting) |  |  |  |  |  |  |
| Others |  | -0.09 (0.28) | -0.27 (0.28) |  | 0.01 (0.40) | 0.26 (0.40) |
| Household income  (ref. highest quartile) |  |  |  |  |  |  |
| 2^nd^ highest |  | -0.44 (0.38) | -0.30 (0.37) |  | 0.17 (0.40) | 0.26 (0.39) |
| 2^nd^ lowest |  | -0.24 (0.38) | -0.29 (0.37) |  | 0.57 (0.44) | 0.37 (0.43) |
| Lowest quartile |  | 0.01 (0.39) | -0.06 (0.38) |  | 0.48 (0.49) | 0.28 (0.49) |
| Economic difficulties  (ref. no) |  |  |  |  |  |  |
| Occasional |  | 0.30 (0.26) | 0.27 (0.25) |  | 0.42 (0.36) | 0.15 (0.35) |
| Frequent |  | 1.16 (0.39)* | 1.06 (0.39)* |  | 1.76 (0.57)* | 1.35 (0.59)* |
| Fruit and vegetable consumption  (ref. daily) |  |  |  |  |  |  |
| Non-daily |  |  | 1.01 (0.27)* |  |  | 0.46 (0.33) |
| Leisure-time physical activity (ref. vigorously active) |  |  |  |  |  |  |
| Moderately active |  |  | 1.05 (0.28)* |  |  | 0.91 (0.36)* |
| Inactive |  |  | 1.52 (0.30)* |  |  | 1.96 (0.39)* |
| Sleep problems (ref. no) |  |  |  |  |  |  |
| Occasional |  |  | -0.42 (0.33) |  |  | -0.24 (0.41) |
| Frequent |  |  | -0.13 (0.47) |  |  | 0.40 (0.56) |
| Current smoker (ref. no) |  |  |  |  |  |  |
| Yes |  |  | -0.72 (0.26)* |  |  | -0.83 (0.37)* |
| Physical health functioning (cont.) |  |  | -0.08 (0.02)* |  |  | -0.08 (0.02)* |
| Mental health functioning (cont.) |  |  | 0.002 (0.01) |  |  | 0.01 (0.02) |
| Intercept | 16.56 (3.30)* | 15.44 (3.34)* | 17.48 (3.66)* | 16.84 (7.00)* | 17.50 (7.06)* | 22.38 (7.31)* |

^a^ Unadjusted model. ^b^ Model 1 + marital status, household income, and economic difficulties. ^c^ Model 2 + fruit and vegetable consumption, leisure-time physical activity, sleep problems, smoking, physical health functioning, and mental health functioning. Abbreviations: SE = standard error, SEP = socioeconomic position. * *p*-value < 0.05.

**Table S4.** Body mass index estimates from mixed-effects linear regression for fixed-effect components among women and men from the younger (birth years 1950–1962) and older (birth years 1940‒1947) birth cohorts: complete case analysis, unadjusted models (Model 1). Beta coefficients with standard errors in parentheses are shown.

|  | **Women ^a^** | | | **Men ^b^** | | |
| --- | --- | --- | --- | --- | --- | --- |
|  | **All** | **Younger birth cohort** | **Older birth cohort** | **All** | **Younger birth cohort** | **Older birth cohort** |
| **Fixed effects, β (SE)** |  |  |  |  |  |  |
| Age | 0.39 (0.05)* | 0.31 (0.08)* | 0.24 (0.19) | 0.28 (0.08)* | 0.31 (0.14)* | 0.23 (0.23) |
| Age^2^ | -0.003 (0.000)* | -0.002 (0.001)* | -0.001 (0.001) | -0.002 (0.001)* | -0.002 (0.001) | -0.002 (0.002) |
| Social mobility (ref. stable high SEP) |  |  |  |  |  |  |
| Upward mobility | -2.41 (1.94) | -4.84 (2.96) | -4.70 (7.87) | -2.40 (3.09) | -2.77 (5.19) | -7.22 (10.07) |
| Downward mobility | -0.17 (2.08) | -0.88 (3.25) | -7.91 (8.99) | 4.59 (3.58) | 9.61 (5.62) | -17.62 (14.33) |
| Stable low SEP | -3.34 (1.59)* | -2.61 (2.40) | -6.81 (6.50) | -5.99 (2.63)* | -2.07 (4.30) | -9.60 (8.54) |
| Social mobility (ref. stable high SEP)*Age |  |  |  |  |  |  |
| Upward mobility | 0.10 (0.07) | 0.20 (0.11) | 0.17 (0.25) | 0.10 (0.10) | 0.12 (0.19) | 0.25 (0.31) |
| Downward mobility | 0.05 (0.07) | 0.08 (0.12) | 0.28 (0.28) | -0.16 (0.12) | -0.35 (0.21) | 0.53 (0.45) |
| Stable low SEP | 0.18 (0.05)* | 0.15 (0.09) | 0.28 (0.20) | 0.24 (0.09)* | 0.09 (0.16) | 0.35 (0.26) |
| Social mobility (ref. stable high SEP)*Age^2^ |  |  |  |  |  |  |
| Upward mobility | -0.001 (0.001) | -0.001 (0.001) | -0.001 (0.002) | -0.001 (0.001) | -0.001 (0.002) | -0.002 (0.002) |
| Downward mobility | -0.001 (0.001) | -0.001 (0.001) | -0.002 (0.002) | 0.002 (0.001) | 0.004 (0.002) | -0.004 (0.003) |
| Stable low SEP | -0.001 (0.000)* | -0.001 (0.001) | -0002 (0.002) | -0.002 (0.001)* | 0.000 (0.002) | -0.003 (0.002) |
| Intercept | 11.06 (1.45)* | 12.95 (2.19)* | 16.17 (5.99)* | 16.77 (2.24)* | 16.05 (3.69)* | 18.18 (7.30)* |

^a^ *N* = 3,988 for all women, *n* = 2,494 for women from the younger birth cohort, and *n* = 1,494 for women from the older birth cohort. ^b^ *N* = 780 for all men, *n* = 458 for men from the younger birth cohort, and *n* = 412 for men from the older birth cohort. Abbreviations: SE = standard error, SEP = socioeconomic position. * *p*-value < 0.05.

Supplementary figures


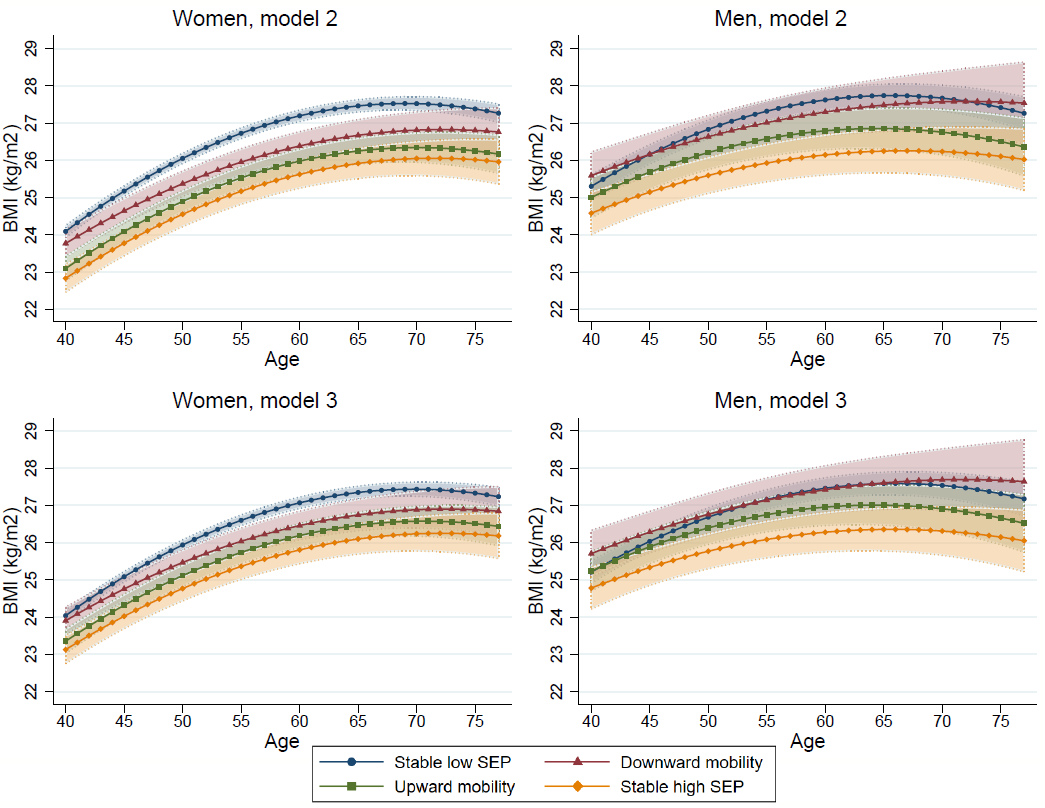


**Fig. S1.** Body mass index (BMI) trajectories by intergenerational social mobility groups over age among women and men. Adjusted Models 2 ^a^ and 3 ^b^: predictive margins—that is, mean BMIs for social mobility groups at each age year—with 95% confidence intervals from mixed-effects linear regression. ^a^ Adjusted for marital status, household income, and economic difficulties. ^b^ Model 2 + fruit and vegetable consumption, leisure-time physical activity, sleep problems, smoking, physical health functioning, and mental health functioning. Abbreviations: SEP = socioeconomic position.


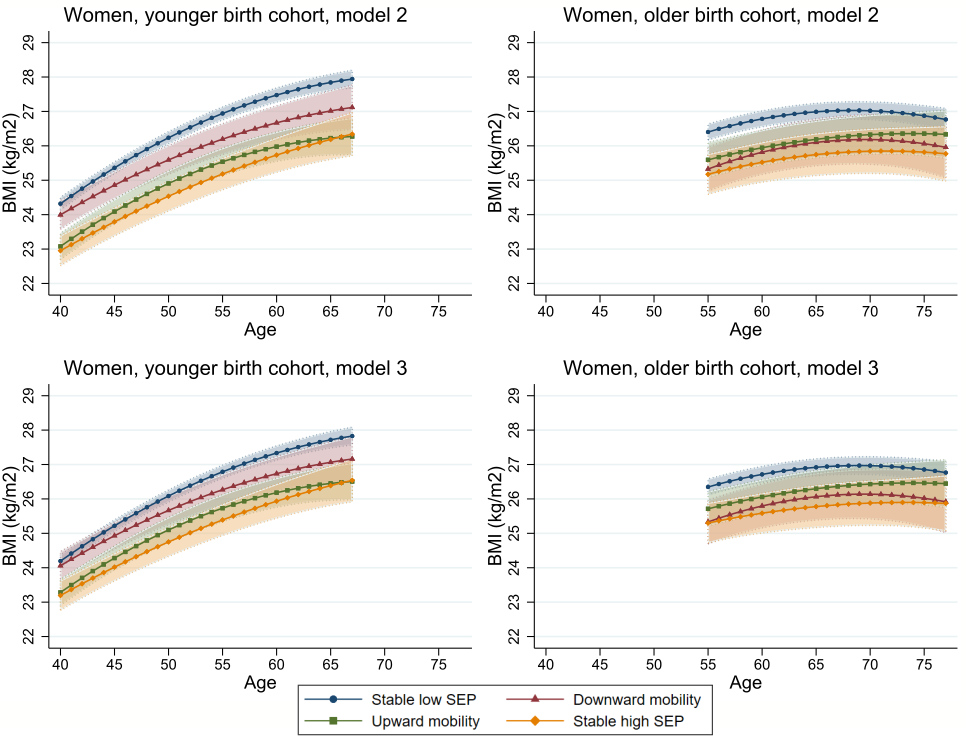


**Fig. S2.** Body mass index (BMI) trajectories by intergenerational social mobility groups over age among women, stratified by birth cohort. Birth years 1950–1962 for younger birth cohort and 1940–1947 for older birth cohort. Adjusted Models 2 ^a^ and 3 ^b^: predictive margins—that is, mean BMIs for social mobility groups at each age year—with 95% confidence intervals from mixed-effects linear regression. ^a^ Adjusted for marital status, household income, and economic difficulties. ^b^ Model 2 + fruit and vegetable consumption, leisure-time physical activity, sleep problems, smoking, physical health functioning, and mental health functioning. Abbreviations: SEP = socioeconomic position.


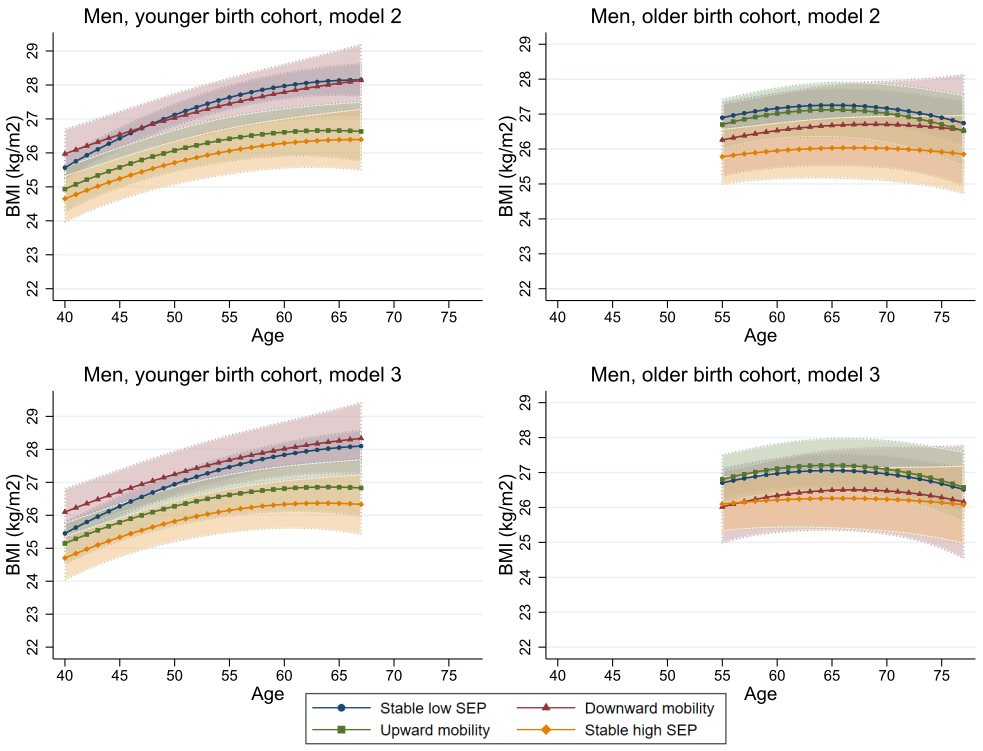


**Fig. S3.** Body mass index (BMI) trajectories by intergenerational social mobility groups over age among men, stratified by birth cohort. Birth years 1950–1962 for younger birth cohort and 1940–1947 for older birth cohort. Adjusted Models 2 ^a^ and 3 ^b^: predictive margins—that is, mean BMIs for social mobility groups at each age year—with 95% confidence intervals from mixed-effects linear regression. ^a^ Adjusted for marital status, household income, and economic difficulties. ^b^ Model 2 + fruit and vegetable consumption, leisure-time physical activity, sleep problems, smoking, physical health functioning, and mental health functioning. Abbreviations: SEP = socioeconomic position.


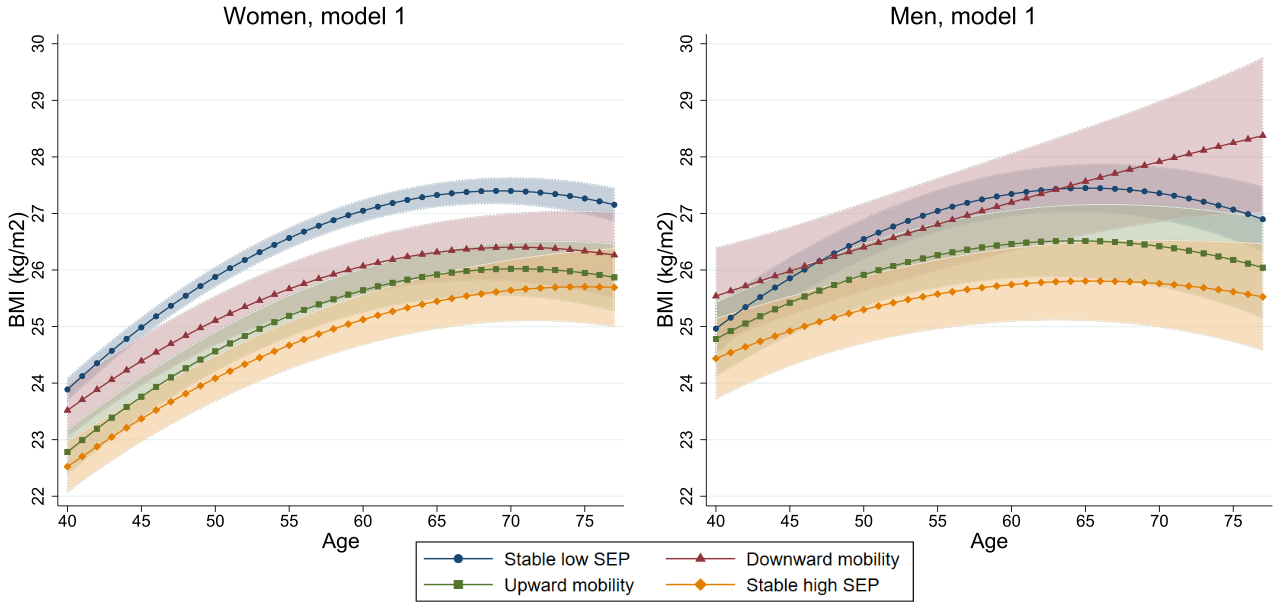


**Fig. S4.** Body mass index (BMI) trajectories by intergenerational social mobility groups over age among women and men: complete case analysis (*n* = 3,988 for women, *n* = 870 for men). Unadjusted models (Model 1): predictive margins—that is, mean BMIs for social mobility groups at each age year—with 95% confidence intervals from mixed-effects linear regression. Abbreviations: SEP = socioeconomic position.


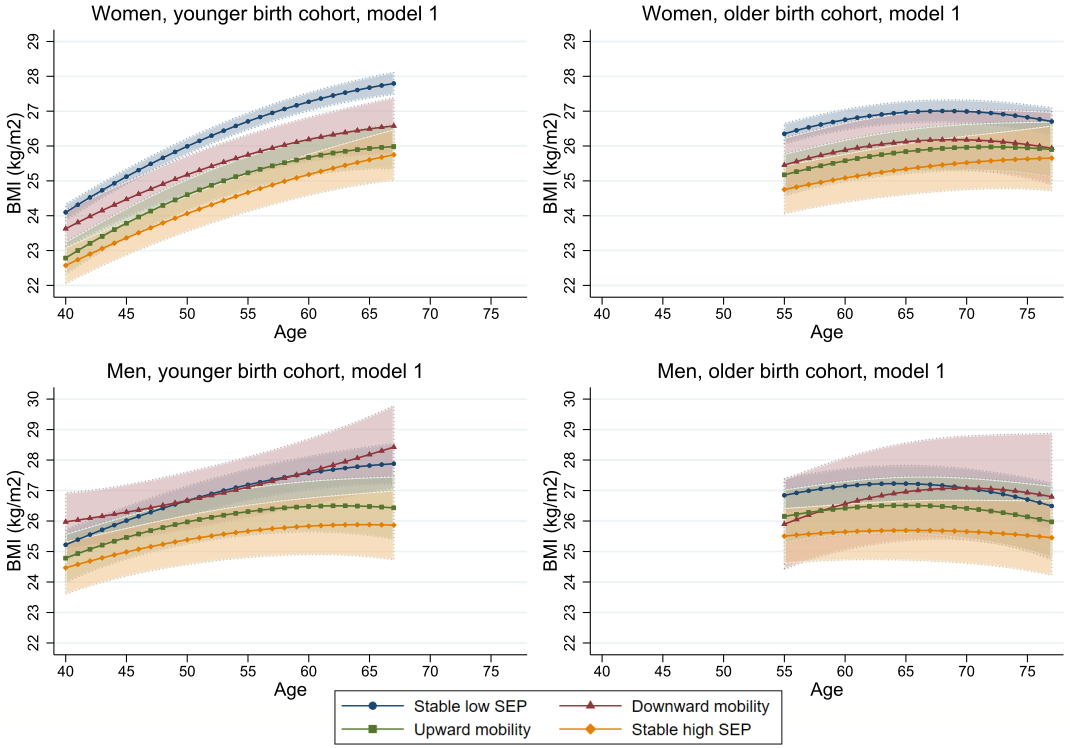


**Fig. S5.** Body mass index (BMI) trajectories by intergenerational social mobility groups over age, stratified by gender and birth cohort: complete case analysis (*n* = 3,988 for women, *n* = 870 for men). Birth years 1950–1962 for younger and 1940–1947 for older birth cohort. Unadjusted models (Model 1): predictive margins—that is, mean BMIs for social mobility groups at each age year—with 95% confidence intervals from mixed-effects linear regression. Abbreviations: SEP = socioeconomic position.


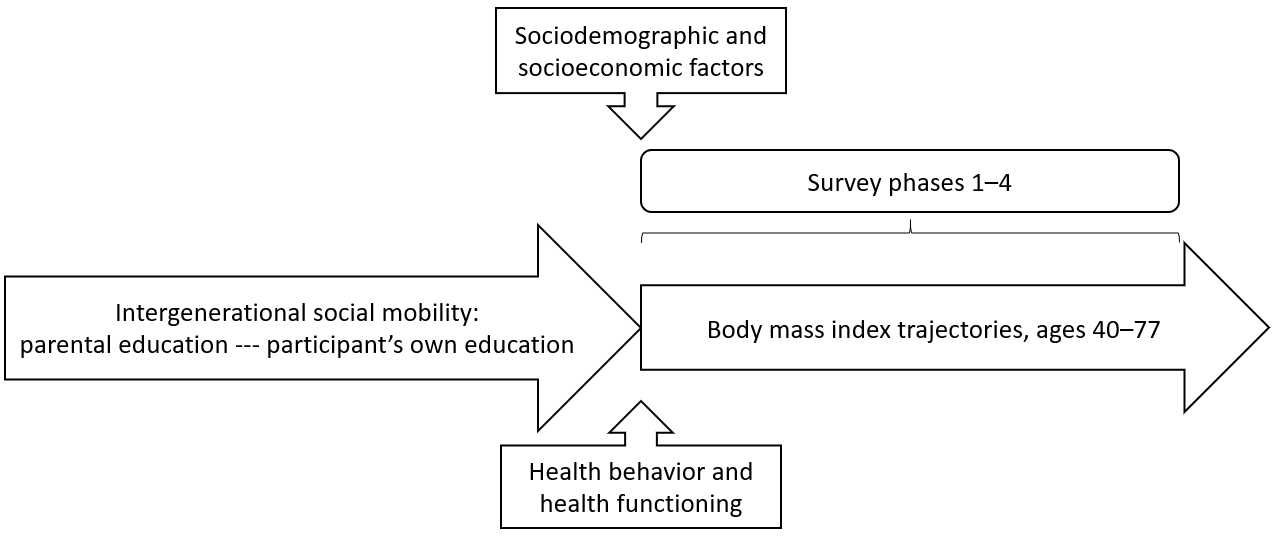


**Fig. S6.** A schematic illustration of the study setting.
